# Partial nephrectomy for small renal tumors may prevent chronic kidney disease

**Published:** 2008

**Authors:** Rajeev Kumar

**Affiliations:** Department of Urology, All India Institute of Medical Sciences, Ansari Nagar, New Delhi - 110 029, India. E-mail: rajeev02@gmail.com

## SUMMARY

The authors from the Department of Urology, University of Texas Southwestern Medical Center at Dallas examined the records of 242 patients who underwent treatment for renal masses < 4 cm in size with a normal contralateral kidney at their center between 1995 and 2005. Among these 242 patients, 86, 85, and 71 were treated with radio frequency ablation, partial nephrectomy, and radical nephrectomy, respectively. At baseline, 26.7% patients, equally distributed among the three treatment arms had stage 3 chronic kidney disease (glomerular filtration rate <60 ml per minute per 1.73 m^2^). Three years following treatment, 95.2, 70.7, and 39.9%, patients, respectively (*P* < 0.001), were likely to have a GFR greater than 60 ml/min. Radical nephrectomy was an independent risk factor for the development of stage 3 chronic kidney disease.

## COMMENTS

Over the last decade, nephron sparing surgery has progressively been established as the treatment of choice for localized renal cancer. It has been shown to be as effective, oncologically, as the more destructive radical nephrectomy. Despite evidence to this effect, nephron sparing surgery continues to be offered to far fewer patients than those who may actually benefit from it. A review of the SEER database found that only 42% patients with tumors <2 cm underwent partial nephrectomy and an even smaller 20% received this treatment if their tumor was between 2 and 4 cm.[1] These findings were in agreement with those of the Nationwide Inpatient Sample that found that despite an increase in the detection of small renal tumors, nearly 90% of all surgeries for renal cancer were radical nephrectomies.[2]

There are probably two major reasons for this dichotomy. Both among patients and their treating physicians, the fear of cancer, as a disease, probably overshadows the fear generated by most other supposedly benign conditions. At every level of medical education, it is taught that surgery is the treatment of choice for kidney cancer and the definitive therapy is radical nephrectomy. Often patients who are referred to urologists for kidney cancer have already been counseled for radical nephrectomy and are likely to consider this a more definitive form of therapy than partial nephrectomy.

The second reason for more radical nephrectomies instead of partial nephrectomies probably lies in the technical simplicity of a radical nephrectomy. Radical nephrectomy is associated with fewer complications and lower blood loss and, most laparoscopic surgeons would be capable of performing a radical nephrectomy laparoscopically but not a partial nephrectomy.

The current manuscript should be an eye-opener for physicians who handle such patients. It highlights two important aspects of this disease. The first is the relatively high prevalence of CKD-III among patients with renal tumors. At 27%, this is higher than the general population. Further, this is unlikely to be diagnosed during a routine renal function evaluation since the blood urea and creatinine values would be normal. The second issue is the high rate of progression to CKD-III even among the patients who did not have a low GFR prior to surgery. This decline was the highest among the patients undergoing a radical nephrectomy.

Chronic kidney disease, though benign, has a socially and economically malignant potential in our country where <2% patients would have medical insurance. It thus behooves us in particular to be careful about selection of our patients for radical nephrectomy just because the other kidney and the renal function look ‘normal’. If we accept that the other kidney is in fact compromised in patients with this disease at an older age, the indication for partial nephrectomy would become absolute rather than relative.
